# Rupture risk assessment for AComA aneurysms with morphological, hemodynamic and structural mechanical analysis

**DOI:** 10.1371/journal.pone.0331297

**Published:** 2025-09-02

**Authors:** Jozsef Nagy, Nico Stroh-Holly, Wolfgang Fenz, Stefan Thumfart, Julia Maier, Zoltan Major, Harald Stefanits, Maria Gollwitzer, Johannes Oberndorfer, Vanessa Mazanec, Michael Giretzlehner, Michael Sonnberger, Philip Rauch, Andreas Gruber, Matthias Gmeiner

**Affiliations:** 1 eulerian-solutions e.U. Leonfeldnerstraße 245, Linz, Austria; 2 Department of Neurosurgery, Kepler University Hospital, Johannes Kepler University Linz, Linz, Austria; 3 RISC Software GmbH, Unit Medical Informatics, Hagenberg, Austria; 4 Johannes Kepler University Linz, Institute of Polymer Product Engineering, Linz, Austria; 5 Institute of Neuroradiology, Kepler University Hospital, Johannes Kepler University, Linz, Austria; 6 Clinical Research Institute for Neuroscience, Johannes Kepler University Linz, Linz, Austria; UCSF: University of California San Francisco, UNITED STATES OF AMERICA

## Abstract

**Introduction:**

The Anterior Communicating Artery complex (AComA) is one of the most common intracranial aneurysms locations. Accurate rupture risk assessment in patients with cerebral aneurysms is essential for optimizing treatment decisions. Computational fluid dynamics has significantly advanced insight into aneurysmal hemodynamics. Many studies concentrate predominantly on blood flow patterns, frequently neglecting the biomechanical properties of the aneurysm wall. Fluid-structure interaction analysis combines hemodynamic behavior with wall mechanics, potentially facilitating a more thorough evaluation of rupture risk assessment.

**Methods:**

In this study, we employed advanced techniques to investigate several single and composite parameters to predict the rupture risk of AComA aneurysms in a cohort of 150 patients treated at the Kepler University Hospital in Linz, Austria. For this reason, clinical, morphological, hemodynamic, and structural mechanical parameters were assessed.

**Results:**

A subsequent workflow analysis, consisting of comparative analysis, collinearity analysis, predictive modeling, composite parameter, performance evaluation, and internal threshold validation, revealed the Gaussian curvature GLN (AUC = 0.91) with a sensitivity of 0.93 and specificity of 0.83 as a best-performing single parameter for aneurysm rupture prediction. Composite parameters like WGD (combination of wall shear stress, Gaussian curvature, and wall displacement) achieved an AUC of 0.89, and WG (combination of wall shear stress and Gaussian curvature) an AUC of 0.88. An internal validation with 25 independent ruptured aneurysms was performed, and the previous results were confirmed with very high sensitivity values of 0.92 for GLN.

**Conclusion:**

Our findings indicate that the investigated morphological, hemodynamic, and structural, mechanical parameters could provide a potential tool for evaluating rupture risk for AComA aneurysms. The single morphological parameter GLN offers, followed by composite parameters WGD and WG, excellent prediction power for the aneurysm rupture state, as confirmed by internal validation. Further studies are warranted to evaluate the prospective clinical application of these parameters.

## 1. Introduction

The intracranial aneurysms (IA) affect approximately 2–5% [1] of the general population. One of the most common locations for IAs is the Anterior Communicating Artery complex (AComA), with a proportion of about 37% [2].

The primary concern and undesirable consequence of IAs is their rupture risk. Aneurysm rupture leads to subarachnoidal hemorrhage (SAH), which is mainly associated with a fatal outcome and very high morbidity and mortality [3]. Recent studies showed that in about 25% of SAH cases, patients die suddenly before reaching the hospital [4,5].

Rupture risk can depend on the aneurysm location [6]. It is well-known that AComA aneurysms are more likely to rupture than other IAs [7,8] and, therefore, they are one of the most frequently treated aneurysms [9].

Because of the increased availability of cranial imaging, like magnetic resonance imaging (MRI) or computed tomography (CT), the detection rate of unruptured intracranial aneurysms has increased considerably over the last few years. This fact leads to an evaluation of whether these aneurysms can be considered stable and can be observed over a certain follow-up period or must be treated [10].

Various risk factors have been shown to impact aneurysms’ growth and rupture risk. Clinical parameters, like age, sex, or smoking habits or morphological factors, like the aneurysm shape, aspect ratio (AR), or size ratio (SR) and hemodynamic factors, like wall shear stress (WSS) or oscillatory shear index (OSI) are often considered to be risk factors [11–13].

Because of the increased availability of cranial imaging, like MRI or CT, the detection rate of unruptured IAs has increased considerably over the last few years. This fact leads to an evaluation of whether these aneurysms can be considered stable and can be observed over a certain follow-up period or must be treated [10].

Different treatment modalities are feasible for AComA aneurysms, such as microsurgical clipping or endovascular approaches, like coiling or endovascular devices [14,15]. Each treatment modality has its particular advantages and procedural risks, and the choice of treatment modality should be made interdisciplinary and case-related [16].

Although microsurgical clipping has consistently achieved excellent aneurysm occlusion and neurological outcome rates [17], endovascular techniques have recently undergone significant improvements. Zandpazandi et al. demonstrated that intraprocedural rupture rates of very small aneurysms have decreased over time [18]. In their series, there was no significant difference in rupture rates between aneurysms <4 mm and ≥4 mm, a finding that likely contributes to enhanced procedural safety and better overall outcomes [18].

In the risk-benefit evaluation, the procedural risk of treating an unruptured IA must be justifiable due to the risk of aneurysm rupture [19]. Substantial indicators for IA rupture risk are necessary to extract unstable and, therefore, dangerous IAs and prioritize treatment [3,19,20].

Computational fluid dynamics (CFD) could be supportive for rupture risk stratification. Research on CFD has elucidated that ruptured aneurysms display different hemodynamic behaviors than unruptured ones [21–24]. These hemodynamic factors exert mechanical stimuli translated into biological signals, potentially promoting aneurysm growth and eventual rupture [25]. Furthermore, the aneurysm’s morphology significantly impacts these hemodynamic parameters [26].

Computational analyses have already addressed the structural mechanics of aneurysms, with a predominant focus on abdominal aneurysms [27,28].

The authors of this study have previously documented a method involving fluid-structure interaction (FSI) analysis [29]. Our current research will employ this FSI methodology to determine hemodynamic and structural mechanical parameters alongside clinical and morphological parameters.

Studies with parallel objectives have primarily concentrated on analyzing average values of ruptured and unruptured aneurysms and conducting multivariate regression analyses with receiver operating characteristic (ROC) curves and area under the curve (AUC) metrics [1,21–24,30]. In this study, we perform similar hemodynamic, structural mechanical, and morphological analyses. We want to identify the highest sensitivity and specificity parameters for the aneurysm rupture state in patients with AComA aneurysms.

## 2. Methods

### 2.1 Patient data

This retrospective study includes patients treated or observed for a single AComA aneurysm at the Department of Neurosurgery, Kepler University Hospital, Linz, Austria.

Patients were categorized into three aneurysm-related cohorts, as previously described for middle cerebral artery aneurysms [31]. The first group comprised patients with ruptured aneurysms treated either endovascularly or microsurgically (Group “ruptured””, n = 58). The second group included patients with unruptured aneurysms electively treated by endovascular or microsurgical methods (Group “unruptured””, n = 52). The dataset for these two groups comprises patients treated in our department between 2016 and 2020. The third group consisted of patients with stable aneurysms who underwent serial follow-up imaging without treatment (Group “stable””, n = 15) in 2023. A stable aneurysm was defined as an untreated, unruptured aneurysm showing no enlargement or morphological changes for at least three years similar to Chung et al [32].

An additional dataset of 25 ruptured aneurysms from 2023 was included for internal threshold validation.

Aneurysm risk factors, including demographic and clinical parameters such as age, sex, smoking status, alcohol abuse, arterial hypertension, and the PHASES Score [33] were retrieved from medical records.

All data were accessed and collected for research purposes between September 1, 2022, and December 31, 2023, after obtaining a positive ethics approval from the local ethics committee (see Chapter “Ethical considerations”).

### 2.2 Hemodynamic and structural mechanical modeling

Digital subtraction angiography (DSA), Computed tomography angiography (CT-A) and Time-of-flight-MRI were used as input for geometry creation and subsequent CFD and FSI simulations. The hemodynamic and structural mechanical modeling followed the methodology previously described for middle cerebral artery aneurysms: [31].

A finite volume solver [29], based on the CFD tool OpenFOAM [34], in conjunction with the FSI library solids4Foam [29,35–37], is employed to solve the unsteady equations during the simulation process numerically. Hemodynamic modeling is governed by mass and momentum conservation principles, using the continuity equation and the Navier-Stokes equations. Newton’s second law is utilized for structural mechanical modeling. Numerical equations were discretized using second-order accuracy, except for time discretization, which employed a first-order Euler scheme. The preconditioned conjugate gradient solver was used to solve the discretized matrix equations, with a convergence tolerance of 1E-6 for fluid dynamic equations and 1E-9 for structural mechanical equations. These equations are solved sequentially and coupled through FSI boundary conditions (FSI tolerance of 1E-5). Details on the simulation method, including experimental setup validation, can be found here [29]. A numerical convergence analysis, which included evaluating time step size and mesh independence, was conducted, and the results are published in [37]. Simulations were executed using four CPU cores in parallel on a development laptop equipped with an i9-11900K processor at 3.50GHz and 64 GB of RAM. Typical simulation runtimes varied from approximately 5–45 minutes.

The fluid dynamic boundary conditions at the inflow are defined by a pulsatile flow profile characterized by a temporal velocity curve derived from published data [38]. The outflow conditions are set according to time-dependent values defined by experimental pressure measurements [39]. A no-slip boundary condition is assumed along the interior vessel walls. Blood is modeled as a Newtonian fluid, with viscosity set at 0.04 Poise and a constant density of 1.06 g/cm³. The influence of non-Newtonian effects on the viscosity was negligible [37]. The simulation simulates a single cardiac cycle of 1 second, corresponding to a heart rate of 60 beats per minute, and is discretized into 100 temporal increments.

In the solid vessel wall with a constant wall thickness of 0.2 mm [40], boundary conditions are imposed by fixing the vessel walls at the inflow and outflow locations. The structural properties of the vascular tissue are approximated using linear elastic material behavior, with a Young’s modulus of 2.49 MPa and a Poisson’s ratio of 0.49, reflecting the relative incompressibility and stiffness of arterial tissue [40].

### 2.3 Investigated parameters

Hemodynamic parameters analyzed included wall shear stress (WSS), oscillatory shear index (OSI), vorticity, and pressure [12,13,21–26,41]. Structural mechanical parameters included wall displacement (D) and equivalent stress (also known as von Mises stress – MISES). Morphological parameters assessed were size (S), aspect ratio (AR), size ratio (SR), volume to ostium ratio (VOR), volume (V), and L2 norm of Gaussian curvature (GLN) are also examined (see Table 1).

**Table 1:** Morphological, hemodynamic, structural mechanical, and derived parameters overview.

Parameter Type	Parameter	Description
**Morphological**	Size (S)	Overall size of the aneurysm
	Aspect Ratio (AR)	Ratio of aneurysm height to neck width
	Size Ratio (SR)	Ratio of aneurysm size to parent vessel diameter
	Volume to ostium ratio (VOR)	Ratio of aneurysm volume to neck surface area
	Volume (V)	Aneurysm volume
	L2 norm of the Gaussian Curvature (GLN)	Measure of curvature considering both principal curvatures [6,22]
**Hemodynamic**	Maximum and Average Wall Shear Stress (WSS_max_, WSS_av_)	Maximum and average shear stress on the aneurysm wall
	Maximum and Minimum Oscillatory Shear Index (OSI_max_, OSI_av_, OSI_min_)	Maximum, average and minimum oscillatory shear index indicating disturbed flow
	Maximum and Minimum Pressure (Pressure_max_, Pressure_av_, Pressure_min_)	Maximum, average and minimum pressure
**Structural Mechanical**	Maximum Wall Displacement (D_max_)	Maximum displacement of the aneurysm wall
	Maximum and Average Von Mises Stress (MISES_max_, MISES_av_)	Maximum and average stress according to von Mises criteria

In this study, a time-dependent pressure profile is utilized, as reported by Blanco et al. [39]. Pressure results are given relative pressure in Pa to the minimum pressure available.

Maxima, minima, and average values of quantities are evaluated according to their availability in simulation post-processing. For the measures of maximum wall shear stress and wall displacement, the relative value is calculated as the ratio of the value in the aneurysm sac (e.g.,. mean value) to the value in the parent vessel.


$${\text{Parameter}}_{\text{rel}}\;=\;{\text{Parameter}}_{\text{aneurysm}}/{\text{Parameter}}_{\text{parent}-\text{vessel}}$$

### 2.4 Statistical analysis workflow

**Fig 1:**
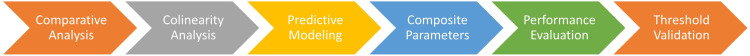
Workflow of the series of statistical analyses performed.

The analysis workflow (Fig 1) involves multiple steps:

1. Comparative analysis:

Descriptive statistics, including frequencies and mean values, were calculated overall and within the groups (ruptured, unruptured, and stable). Mann-Whitney U-tests are used to assess differences between the groups for parameters without normal distributions. Non-significant parameters are excluded.

Given the absence of significant differences between ruptured and unruptured treated aneurysms across several analyzed parameters, subsequent analyses will focus on comparing ruptured and stable aneurysms.

2. Collinearity analysis:

Collinearity is evaluated to remove linearly dependent parameters. Parameters exhibiting statistically significant differences between mean values in U-tests are further investigated for collinearity to guarantee our investigated variables’ linear independence. If the Pearson coefficients between parameters are high (> 0.75), parameters are excluded from further investigation.

3. Predictive modeling:

After a collinearity check of the ruptured and stable aneurysms, univariate and subsequent multivariate regression analyses identify independent predictors of aneurysm rupture.

4. Composite parameters:

These independent predictors of aneurysm rupture, identified as significant parameters in the multivariate analyses, are subsequently combined into composite parameters to enhance sensitivity and specificity further.

5. Performance evaluation:

Receiver operating characteristic (ROC) curves and area under the curve (AUC) values are calculated to assess the predictive performance of the single and composite parameters. Threshold values are optimized for sensitivity and specificity.

6. Threshold validation:

The internal threshold validation is performed using an additional dataset of 25 ruptured aneurysms. The sensitivity of the parameters is calculated (Identifying ruptured aneurysms correctly as such).

### 2.5 Ethical considerations

This study was approved by the local ethics committee (Ethikkommission der medizinischen Fakultät der Johannes Kepler Universität; EK Nr: 1129/2022). All procedures performed were in accordance with the ethical standards of the institutional and national research committee, and with the 1964 Helsinki Declaration and its later amendments or comparable ethical standards.

## 3. Results

### 3.1 Demographic and clinical parameters

In 125 patients with AComA aneurysms, the mean age of the cohort was 56.8 years and 53 patients (42.4%) were female. In 125 patients with AComA aneurysms, the mean age of the cohort was 56.8 years, and 53 patients (42.4%) were female. The overall mean PHASES score was 5.45, with no significant differences observed among the groups. Chronic alcohol abuse was present in 10 patients (8%) overall, with no significant variation between groups. Similarly, 19 patients (15.2%) were smokers, but no intergroup differences were noted. Other established risk factors, such as age (56.8 years), female sex (42.4%), and arterial hypertension (42.4%), also showed no significant differences between the groups. The demographic and clinical characteristics of the study population are summarized in Table 2.

**Table 2:** Descriptive analysis of demographic and clinical parameters with group differences assessed by the Mann-Whitney U test. AHT = Arterial hypertension, vs = versus, SD = standard deviation.

	Groups (mean values)				Group comparison (p-value)		
	All (n = 125)	Ruptured (n = 58)	Unruptured (n = 52)	Stable (n = 15)	Ruptured Vs. Unruptured	Ruptured vs. Stable	Unruptured vs. Stable
**Age** (<70 = 0 and >70 = 1)	0.180	0.151	0.214	0.25	0.5241	0.4605	0.7706
**Alcohol**	10 (8%)	3	6	1	0.1974	0.8303	0.6068
**Smoking**	19 (15.2%	8	9	2	0.5203	0.7797	0.7362
**Sex (female)**	53 (42.4%)	24	21	8	0.5393	0.6530	0.4199
**AHT**	53 (42.4%)	28	19	6	0.3746	0.7734	0.7991
**PHASES** (mean ± SD)	5.45 ± 1.89	5.49 ± 1.71	5.41 ± 2.09	5.19 ± 1.42	0.4293	0.6803	0.9255

### 3.2 Hemodynamic, structural mechanical, and morphological parameters

Hemodynamic, morphological, and structural mechanical parameters were analyzed (Table 3a–3c). Among hemodynamic parameters, WSS_rel,max_, WSS_max_, and OSI_min_ were significantly elevated in ruptured aneurysms compared to stable aneurysms (p < 0.05). Other hemodynamic measures, WSSav, OSImax, and all Pressure values, did not exhibit significant intergroup differences (Table 3.a)

**Table 3:** Results from statistical U-test Analysis for investigating hemodynamic (3.a.), structural mechanical (3.b.), and morphological parameters (3.c.).

	Groups (mean values)			Group Comparison (p-value)		
	Ruptured	Unruptured	Stable	Ruptured vs Unruptured	Ruptured vs Stable	Unruptured vs Stable
**a.Hemodynamic Variables**						
WSS_rel,max_ [Pa]	0.514	0.363	0.260	0.2467	0.0021	0.0193
WSS_max_ [Pa]	6.127	4.231	2.590	0.3821	0.0040	0.0114
WSS_av_ [Pa]	0.244	0.223	0.260	0.4573	0.3090	0.4653
OSI_max_ [-]	0.370	0.349	0.289	0.2186	0.0649	0.0965
OSI_av_ [-]	0.062	0.056	0.062	0.2467	0.7892	0.2845
OSI_min_ [-]	1.536E-03	2.974E-03	7.011E-03	0.6993	0.0013	0.1125
Pressure_max_ [kPa]	11.115	9.236	9.169	0.3803	0.5890	0.2822
Pressure_av_ [kPa]	5.784	5.621	5.530	0.6897	0.5832	0.5384
Pressure_min_ [kPa]	3.712	3.944	3.969	0.3245	0.5439	0.2349
**b.Structural mechanical Variables**						
D_rel,max_ [-]	2.421	2.037	1.445	0.0473	<0.001	0.0032
MISES_max_ [kPa]	250.3	218.7	174.1	0.1713	0.004	<0.001
MISES_av_ [kPa]	75.27	70.53	60.67	0.5276	0.1210	0.3935
**c.Morphological Variables**						
S [mm]	6.929	6.833	4.448	0.5355	<0.001	0.0018
AR [-]	1.092	0.957	0.655	0.1015	<0.001	0.0013
SR [-]	3.711	3.295	2.157	0.1482	<0.001	<0.001
VOR [-]	4.738	4.516	1.474	0.6994	<0.001	<0.001
V [m^3^]	75.29	96.44	18.63	0.4633	<0.001	<0.001
GLN [-]	2.164	1.651	0.927	0.0015	<0.001	<0.001

Structural mechanical parameters, including D_rel,max,_ and MISES_max_, showed not only significant differences between ruptured and stable aneurysms (D_rel,max_, p=<0.001; MISES_max_, p = 0.004), but also between unruptured and stable aneurysms (D_rel,max_, p=<0.0032; MISES_max_, p=<0.001). MISES_av_ showed no significant intergroup differences (Table 3.b).

All morphological parameters – S (p=<0.001), AR (p=<0.001), SR (p=<0.001), VOR (p=<0.001), V (p=<0.001), GLN (p=<0.001) – demonstrated significant differences between ruptured and stable aneurysms (Table 3.c).

As outlined in the analysis workflow and given the lack of significant differences between ruptured and unruptured treated aneurysms across several analyzed parameters, subsequent analyses focused exclusively on comparing ruptured and stable aneurysms. As a result, only parameters with statistically significant differences between ruptured and stable aneurysms and only patients of the ruptured and stable aneurysm cohorts were included in the subsequent steps.

**Fig 2:**
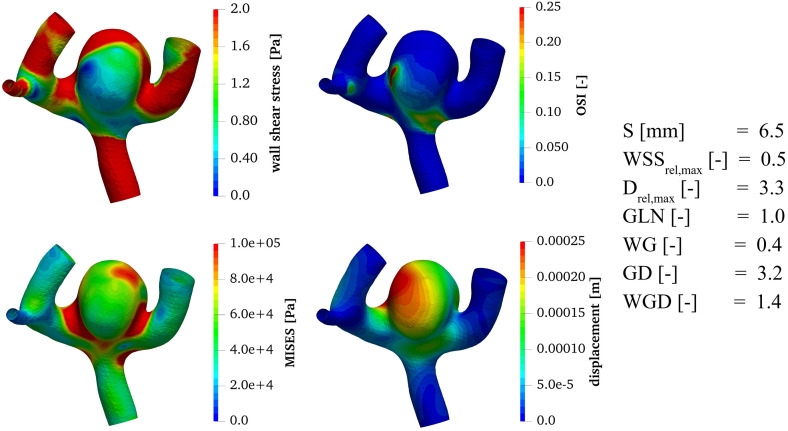
Exemplary results of WSS, OSI, displacement and MISES in a ruptured aneurysm; on the left selected morphological, hemodynamic and structural mechanical parameters of this aneruysm are given.

Fig 2 shows results of WSS, OSI, displacement as well as MISES stress in a selected ruptured aneurysm.

### 3.3 Collinearity analysis

The Pearson correlation coefficient revealed a strong correlation between the hemodynamic parameters WSS_max_ and WSS_rel,max_ (ρ = 0.989), see Table 4. Among the morphological parameters, several high collinearities were identified, including between S and SR (ρ = 0.799), S and V (ρ = 0.823), AR and VOR (ρ = 0.767), and VOR and V (ρ = 0.793). Notably, GLN was the only morphological parameter not exhibiting significant correlation (ρ > 0.75) with other morphological measures. No collinearity was observed among the structural mechanical parameters, as determined by the Pearson correlation coefficients.

**Table 4:** Pearson coefficients of hemodynamic (a), structural mechanical (b), and morphological (c) parameters.

(a)	WSS_rel,max_		WSS_max_				OSI_min_
**WSS**_**rel,max**_	1		0.989				−0.016
**WSS**_**max**_	0.989		1				−0.016
**OSI**_**min**_	−0.016		−0.016				1
b)	**D**_**rel,max**_				**MISES**_**max**_		
**D**_**rel,max**_	1				0.234		
**MISES**_**max**_	0.234				1		
c)	**S**	**AR**	**SR**	**VOR**		**V**	**GLN**
**S**	1	0.502	0.799	0.735		0.823	0.683
**AR**	0.502	1	0.448	0.767		0.387	0.448
**SR**	0.799	0.448	1	0.491		0.661	0.580
**VOR**	0.735	0.767	0.491	1		0.793	0.451
**V**	0.823	0.387	0.661	0.791		1	0.445
**GLN**	0.683	0.448	0.580	0.451		0.445	1

As a result, only non-collinear parameters WSS_rel,max_, OSI_min_, D_rel,max_, MISES_max_, and GLN were included in the subsequent steps.

### 3.4 Predictive modeling

Univariate analysis (Table 5) identified two hemodynamic parameters, WSS_rel,max_, (p = 0.0078) and OSI_min_ (p = 0.0232),) significantly associated with aneurysm rupture. Also, two structural mechanical parameters, D_rel,max_ (p = 0.0029) and MISES_max_ (p = 0.0161),) and the morphological parameter GLN (p=<0.001) showed significant association with aneurysm rupture. Multivariate regression analysis (Table 5) reduced the set of significant predictors to three key parameters: WSS_rel,max_ (p = 0.00283), D_rel,max_ (p = 0.0062), and GLN (p = 0.0326). As a result, these three parameters were selected for further sensitivity analyses.

**Table 5:** Odds ratio (OR), 95% confidence interval (95%CI), and p-value in univariate and multivariate regression analysis between ruptured and stable aneurysms for hemodynamic, structural mechanical, and morphological parameters. RA = Regression Analysis, OR = Odds Ratio.

	Univariate RA			Multivariate RA		
	OR	95%CI	p-Value	OR	95%CI	p-Value
**WSS**_**rel,max**_	2.21	1.41-3.47	0.0078	2.33	1.31-3.54	0.00283
**OSI**_**min**_	1.45	1.14-1.91	0.0232	1.43	0.97-3.98	0.0766
**D**_**rel,max**_	1.66	1.47-1.98	0.0029	1.70	1.52-2.03	0.0062
**MISES**_**max**_	2.55	1.88-3.67	0.0161	2.61	1.95-3.73	0.079
**AR**	2.79	2.05-4.06	0.0015	4.72	3.45-7.83	0.2882
**SR**	2.80	2.01-3.73	0.0164	4.13	2.97-6.91	0.2711
**V**	1.06	1.03-1.11	0.0096	1.04	1.01-1.08	0.8870
**GLN**	2.64	1.62-4.07	<0.001	2.76	1.69-4.01	0.0326

### 3.5 Composite parameters

Three additional composite parameters – WG (WSS_rel,max_ * GLN), GD (GLN * D_rel,max_), and WGD (WSS_rel,max_ * GLN * D_rel,max_) – were derived to improve sensitivity and specificity for rupture prediction. Table 6 lists the composite parameters’ definitions, mean values, and p-values in U-tests.

**Table 6:** Results from statistical U-test analysis for additional parameters.

	Definition (Formula)	Groups (mean values)			Group comparison (p-value)		
		Ruptured	Unruptured	Stable	Ruptured vs Unruptured	Ruptured vs. Stable	Unruptured vs. Stable
**WG**	WSS_rel,max_ * GLN	1.162	0.555	0.287	0.0142	<0.001	<0.001
**GD**	GLN * D_rel,max_	5.409	3.133	1.469	0.0015	<0.001	<0.001
**WGD**	WSS_rel,max_ * GLN * D_rel,max_	3.065	1.240	0.469	0.0070	<0.001	<0.001

### 3.6 Performance evaluation

**Fig 3:**
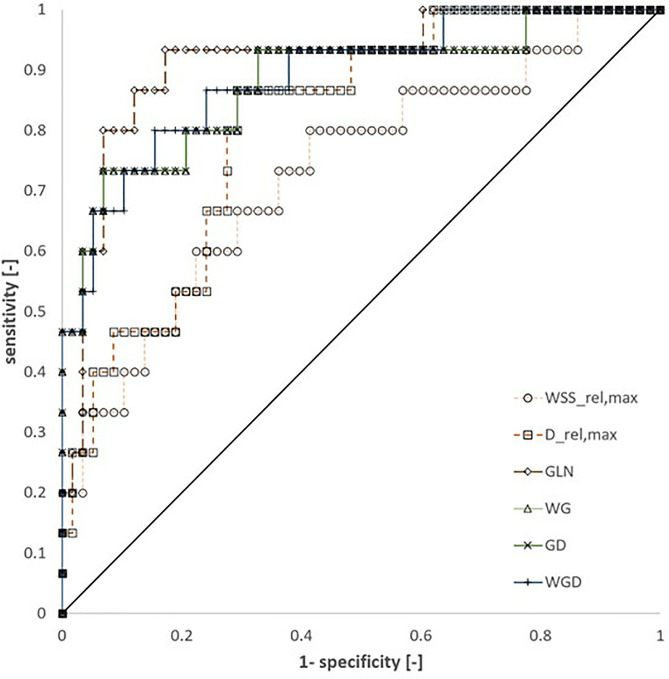
Receiver operating characteristic (ROC) curves for all parameters.

The best-performing single parameter was GLN, achieving an AUC of 0.91, followed by D_rel,max_ (AUC = 0.81). Among the composite parameters, WGD achieved an AUC of 0.89, WG an AUC of 0.88, and GD an AUC of 0.82, as demonstrated in Fig 3 and Table 7. Fig 3 shows the ROC curves in the sensitivity over 1-specificity diagram. Table 7 provides the AUC.

**Table 7:** Area under the curve (AUC) values, sensitivity, specificity, and corresponding threshold values of all remaining parameters.

	AUC [-]	Threshold value	Sensitivity	Specificity
**WSS**_**rel,max**_	0.733	0.3	0.67	0.71
**D**_**rel,max**_	0.810	1.55	0.87	0.71
**GLN**	0.914	1.1	0.93	0.83
**WG**	0.881	0.35	0.87	0.81
**GD**	0.815	1.9	0.80	0.79
**WGD**	0.890	0.5	0.86	0.76

A sensitivity threshold of >0.8 was targeted for clinical application while maintaining acceptable specificity. GLN achieved a high sensitivity of 0.93 and a specificity of 0.83 at a threshold of 1.1. Similarly, the composite parameter WGD demonstrated a strong sensitivity of 0.86 and a specificity of 0.76 at a threshold of 0.5.

### 3.7 Threshold validation

The GLN parameter correctly identified 23 of 25 ruptured aneurysms in a separate independent dataset of 25 ruptured aneurysms, yielding the highest accuracy of 92%. Composite parameters WG, GD, and WGD each correctly identified 21 of 25 cases (84%).%) correctly. By comparison, WSS_rel,max_ correctly identified 18 of 25 cases (72%). Internal validation results are reported in Table 8.

**Table 8:** Internal validation (n = 25) for all remaining parameters, sensitivity of individual parameters, and number of correctly identified aneurysms as ruptured.

	Rupture Prediction (%)
**WSS**_**rel,max**_	18/ 25 (72%)
**D**_**rel,max**_	21/25 (84%)
**GLN**	23/ 25 (92%)
**WG**	21/ 25 (84%)
**GD**	21/ 25 (84%)
**WGD**	21/ 25 (84%)

## 4. Discussion

AcomA aneurysms account for over one-third of all intracranial aneurysms and may exhibit a higher rupture risk than other intracranial locations [2,42,43]. Recently, it has been demonstrated that even small AcomA aneurysms (4–7 mm) can have a significant rupture risk comparable to posterior circulation aneurysms [43]. Consequently, a detailed rupture risk analysis is essential in these patients. This study employed comprehensive FSI techniques to evaluate fluid dynamics, structural dynamics, and morphology to improve rupture risk assessment.

We analyzed 150 AComA aneurysms, revealing significant differences between ruptured and unruptured aneurysms. We propose that comparing morphological, hemodynamic, and structural mechanical parameters across these groups is essential for understanding the factors driving aneurysm rupture versus stability. To address this, we subdivided the unruptured group into two categories: an “unruptured (treated)” group and an “unruptured stable” group [31]. The stable group may enhance clarity for such analysis and serve as a more reliable reference for low rupture risk, as these aneurysms exhibited no changes in size or morphology for at least three years. An exact definition of “stable” aneurysms is not provided in CFD studies. To ensure we analyzed a truly stable interval, we set our cut-off at three years. In other publications, no specific interval was defined [44], or a minimum follow-up imaging interval of twelve months was reported [10,32]. We therefore believe that we have employed a clinically relevant interval for our investigations.

Existing literature indicates that the rupture risk of IAs varies by location [6], with AComA aneurysms displaying a higher rupture risk than aneurysms in other locations [7,8]. Consequently, it is necessary to evaluate rupture risk specifically for AComA aneurysms rather than grouping them with aneurysms from different sites.

Risk analysis is commonly performed using clinical and demographic risk factors, including aneurysm size, family history, genetic predisposition, prior subarachnoid hemorrhage, and modifiable factors such as smoking and hypertension [6,45]. Several scores (e.g., PHASES, ELAPPS, UCAS) have been developed to estimate the risk of aneurysm rupture and growth. However, a prospective multicenter study demonstrated that these tests would not have identified most patients who ultimately experienced rupture [46]. Our results are in line with this study. We did not observe differences regarding the PHASES Score between ruptured and unruptured aneurysms.

In our cohort, the proportion of female patients (42.4%) was lower than in previous reports [43]. Bijlenga and colleagues analyzed 932 patients with AComA aneurysms and found that 71% were female. However, in our study, gender did not influence rupture risk, and no significant differences were observed between groups.

Morphological studies have highlighted the importance of morphologic analysis. Especially aneurysms that deviate from a spherical shape with irregularities may be associated with a higher risk of rupture [25]. Chen et al. analyzed morphological factors and demonstrated that anterior projection and irregular aneurysms were associated with aneurysm rupture in multivariate analysis [42]. Liu et al. developed a decision tree model to assess the rupture risk based on size ratio, flow angle, vessel angle, aneurysm irregularity, and aneurysm size [47].

While most morphological parameters in our study show significant differences between stable aneurysms and the other two cohorts, GLN was the only morphological parameter significantly linked to rupture risk in our multivariate analysis. The results might indicate that GLN is a relevant parameter, potentially aiding in the identification of aneurysms at higher risk for rupture. Aneurysm irregularity can not be precisely defined, whereas GLN can be measured accurately and may provide more reliable results. Recently, we could demonstrate that GLN was even associated with rupture risk in patients with middle cerebral artery aneurysms.

Moreover, an association with aneurysm growth was reported recently [44].

Hemodynamic parameters, such as WSS and oscillatory shear index OSI, have been debated regarding their association with aneurysm rupture [48]. Our study indicates a significant association between WSS and rupture status.

Detmer et al. systematically investigated the association between hemodynamic parameters and aneurysm rupture, demonstrating that hemodynamic and morphological differences between ruptured and unruptured aneurysms are consistent across various anatomical locations [49]. Their analysis revealed that WSS was significantly higher in ruptured aneurysms than in unruptured ones [49]. Similarly, Tian et al. focused specifically on AComA aneurysms. They showed that hemodynamic metrics such as WSS may be associated with rupture risk, and that the combination of hemodynamic and morphological parameters is valuable for distinguishing between ruptured and unruptured aneurysms [50]. These findings for AComA aneurysms have been supported by several subsequent studies, which likewise reported elevated WSS in ruptured AComA aneurysms [51,52]. However, the relationship between WSS and aneurysm rupture appears to be complex and may depend on patient-specific or methodological factors, as findings across studies are not entirely consistent. In contrast, Zhang et al. reported lower WSS values in ruptured aneurysms, based on a cohort of patients with multiple aneurysms located on the same side of the anterior circulation but with differing rupture status [53].

Furthermore, a “high-versus-low wall shear stress” controversy has been introduced, and both high and low WSS may impact aneurysm growth and rupture [13]. Our study showed that even though average WSS values did not show significant differences, maxima of WSS are reported to be at higher values in ruptured aneurysms compared to stable ones. Unruptured aneurysms show a maximum value between these two cohorts. Also, OSI seems to show a significant difference between stable aneurysms and the other two cohorts, aligning with the findings presented in [48].

Structural mechanical parameters such as the equivalent strain of equivalent stress have been investigated in their relationship to aneurysm rupture [40,48]. The strain represents how much a cerebral blood vessel is stretched. The higher the value, the stronger it is stretched (e.g., 0.05 corresponds to 5% stretch). In a study, 51 patients were investigated, and elevated equivalent strains in ruptured aneurysms were reported [40].

In a separate study, the combination of WSS and strain provided better accuracy than a single-factor analysis (WSS or strain alone; see combined analysis below) [54]. In our current work, rather than investigating the stretch in percentage points, we focus on the displacement of the vessel wall compared to the initial state in meters. To have a unitless quantity, we scale the displacement of the aneurysm sac to the displacement of the parent vessel and only analyze the ratio of both. With this, we include the stretch of the aneurysm and analyze the wall movement on and around the aneurysm dome. This relative ratio of maxima of displacement shows the highest significant differences between the cohorts among structural mechanical parameters and elevated sensitivity for rupture risk (0.87).

As reported by Yang et al., the combination of hemodynamic and structural mechanical parameters (WSS and strain) was shown to provide better accuracy [54]. In our work, we similarly define combined best parameters into additional parameters (see Table 6). These parameters show high sensitivity and specificity values regarding rupture risk (see Tables 6 and 7).

### 4.1 Clinical implications

Our findings may improve the clinical practice’s rupture risk stratification for AComA aneurysms. Our results suggest that incorporating hemodynamic and structural mechanical parameters may enhance the predictive accuracy for rupture risk. This could be especially relevant in cases where treatment indication remains ambiguous, such as in small or morphologically irregular aneurysms without other high-risk features. The subdivision of unruptured aneurysms into “treated” and “stable” groups further refines the reference for low-risk aneurysms. Although further prospective validation is needed, integrating FSI-derived parameters into the existing risk assessment framework may support more individualized treatment decisions and ultimately improve patient outcomes.

## 5. Limitations

This study is, however, subject to limitations. A primary constraint is a dependence on assumptions about the artery’s main shape during aneurysm segmentation, restricted by current imaging technologies and resolution capabilities. In the FSI simulations, it is necessary to make assumptions about fixing the geometry. In this study, the blood vessels are fixed at the ends of the inflow and outflow vessels. However, this does not accurately reflect real-life conditions where blood vessels are connected to other vessels. The simulations in this study do not account for possible contact with brain tissue or other vessels either, which could affect the structural mechanical behavior of the aneurysms.

Another limitation of our study is the complexity of AComA aneurysms caused by anatomical variations in the A1 segments, which can significantly influence hemodynamic conditions. Future studies could consider stratifying AComA aneurysms based on A1 dominance or symmetry to better account for these hemodynamic differences.

Additionally, using a constant vessel wall thickness of 0.2 mm, although based on existing literature [40], adds another layer of uncertainty. The empirical data for pulsatile blood flow introduces potential variability, and due to the lack of comprehensive material property data for individual aneurysms, assumed values were used for Young’s modulus and Poisson’s ratio. These necessary approximations limit the precision of our models. Furthermore, the study did not exclude outliers in the multivariate regression analysis, potentially affecting the robustness of our findings.

## 6. Conclusions

This study underscores the importance of advanced morphological, hemodynamic, and structural mechanical analyses in evaluating rupture risk for AComA aneurysms. We demonstrate their potential to facilitate robust cohort stratification by integrating these three parameter sets. For a more rigorous statistical evaluation, we subdivided the unruptured aneurysm group into unruptured but treated and unruptured but untreated (stable). Our findings indicate that clinical parameters alone are insufficient for accurately assessing rupture risk.

We identified parameters such as GLN with a sensitivity as high as 0.91, highlighting their promise for future clinical application. Finally, we confirmed the reliability of these parameters using an independent validation set of 25 aneurysms, demonstrating the robustness of our conclusions. Next, we will conduct multicenter studies to validate and refine our models.

## Abbreviations

AComA: Anterior Communicating Artery
AHT: Arterial Hypertension
AR: Aspect Ratio
AUC: Area Under the Curve
CFD: Computational Fluid Dynamics
CI: Confidence Interval
CT: Computed Tomography
D: Wall Displacement
FSI: Fluid-structure Interaction
GLN: Gaussian Curvature
IA: Intracranial Aneurysm
MISES: Equivalent Stress
MRI: Magnetic Resonance Imaging
OR: Odds Ratio
OSI: Oscillatory Shear Index
RA: Regression Analysis
ROC: Receiver Operating Characteristic
S: Size
SAH: Subarachnoid Hemorrhage
SD: Standard Deviation
SR: Size Ratio
V: Volume
VOR: Volume to Ostium Ratio
vs: Versus
WSS: Wall Shear Stress
